# A Recurrent Small Cell Lung Carcinoma Harboring an EML4–ALK Fusion Mutation with Sustained Response to Ensartinib: A Case Report

**DOI:** 10.3390/curroncol32030163

**Published:** 2025-03-13

**Authors:** Hao Jiang, Tengfei Zhu, Zenghao Chang, Ziyu Liu, Wei Ou, Siyu Wang

**Affiliations:** Department of Thoracic Surgery, Sun Yat-sen University Cancer Center, State Key Laboratory of Oncology in South China, Collaborative Innovation Center for Cancer Medicine, No. 651 Dongfeng East Road, Yuexiu District, Guangzhou 510060, China; jianghao@sysucc.org.cn (H.J.); zhutf@sysucc.org.cn (T.Z.); changch@sysucc.org.cn (Z.C.); liuzy2@sysucc.org.cn (Z.L.); ouwei@sysucc.org.cn (W.O.)

**Keywords:** EML4-ALK fusion, ensartinib, NGS, SCLC, case report

## Abstract

Small cell lung cancer (SCLC) is an aggressive neuroendocrine tumor. Lung cancer patients with ALK and EML4 fusions respond significantly to ALK inhibitors. The EML4-ALK fusion gene mutation is the result of an inversion of chromosome 2, which juxtaposes the 5 end of the EML4 gene with the 3 end of the ALK gene. In SCLC, the frequency of fusion genes is very low, and to the best of our knowledge, only four cases of ALK fusion gene mutations in SCLC have been reported. In this report, we describe the treatment of a 74-year-old female patient with SCLC who developed recurrence of hilar lymph node metastasis three years after surgical resection. Postoperative NGS showed that this patient is a SCLC patient harboring a rare EML4-ALK fusion mutation, and a satisfactory 43-month overall survival (OS) was achieved after treatment with ensartinib targeting the EML4-ALK fusion gene mutation. The ALK-TKI may be a new treatment option for these patients. This article provides a therapeutic reference.

## 1. Introduction

Neuroendocrine tumors account for approximately 20% of all lung cancers, and small cell lung cancer (SCLC), an aggressive cancer of neuroendocrine origin, accounts for approximately 14% of neuroendocrine tumor cases [1]. Characterized by rapid progression, early metastasis, and frequent recurrence, SCLC has a poor prognosis, with 5-year survival rates generally below 7% [2]. The standard treatment for limited-stage SCLC has remained largely unchanged for over two decades, typically involving concurrent chemotherapy with cisplatin and etoposide alongside thoracic radiotherapy [3,4]. However, most patients experience relapse and disease progression within months of initial therapy. Despite advances in cancer treatment, the prognosis for SCLC patients remains dismal, underscoring the urgent need for new therapeutic strategies.

SCLC is frequently characterized by multiple genetic alterations, with the more susceptible genes being TP53, RB1, MYC, PTEN, PI3K, C-KIT, and C-MET [5]. However, mutations involving the EML4-ALK fusion gene, which is typically associated with non-small cell lung cancer (NSCLC), are exceedingly rare in SCLC. The clinical features of lung cancers carrying mutations in the echinoderm microtubule associated protein-like (EML4)-ALK fusion gene include mild or never smoking, younger age, adenocarcinoma with an alveolar pattern or impression ring adenocarcinoma, and lack of EGFR or KRAS mutations [6]. Five ALK tyrosine kinase inhibitors (TKIs) are currently approved by the U.S. Food and Drug Administration (FDA) for the treatment of patients with ALK-positive lung cancer [7]. This fusion gene, resulting from a chromosomal inversion, has shown promising responses to ALK inhibitors in NSCLC, but its role in SCLC remains underexplored [8]. Currently, only a few cases of small cell lung cancer (SCLC) harboring the EML4-ALK fusion gene have been reported, yet the potential of ALK inhibitors to treat these cases remains a topic of significant interest.

This case report describes the treatment of a 74-year-old female patient carrying an EML4-ALK fusion gene mutation in SCLC who developed recurrence of hilar lymph node metastasis three years after surgical resection and was treated with ensartinib targeting the EML4-ALK fusion gene mutation. Through the clinical course of the patient, who experienced a sustained response to targeted therapy, we aim to provide new insights into the feasibility and potential efficacy of ALK inhibitors in treating SCLC with this rare genetic mutation.

## 2. Case Presentation

**Figure 1:**
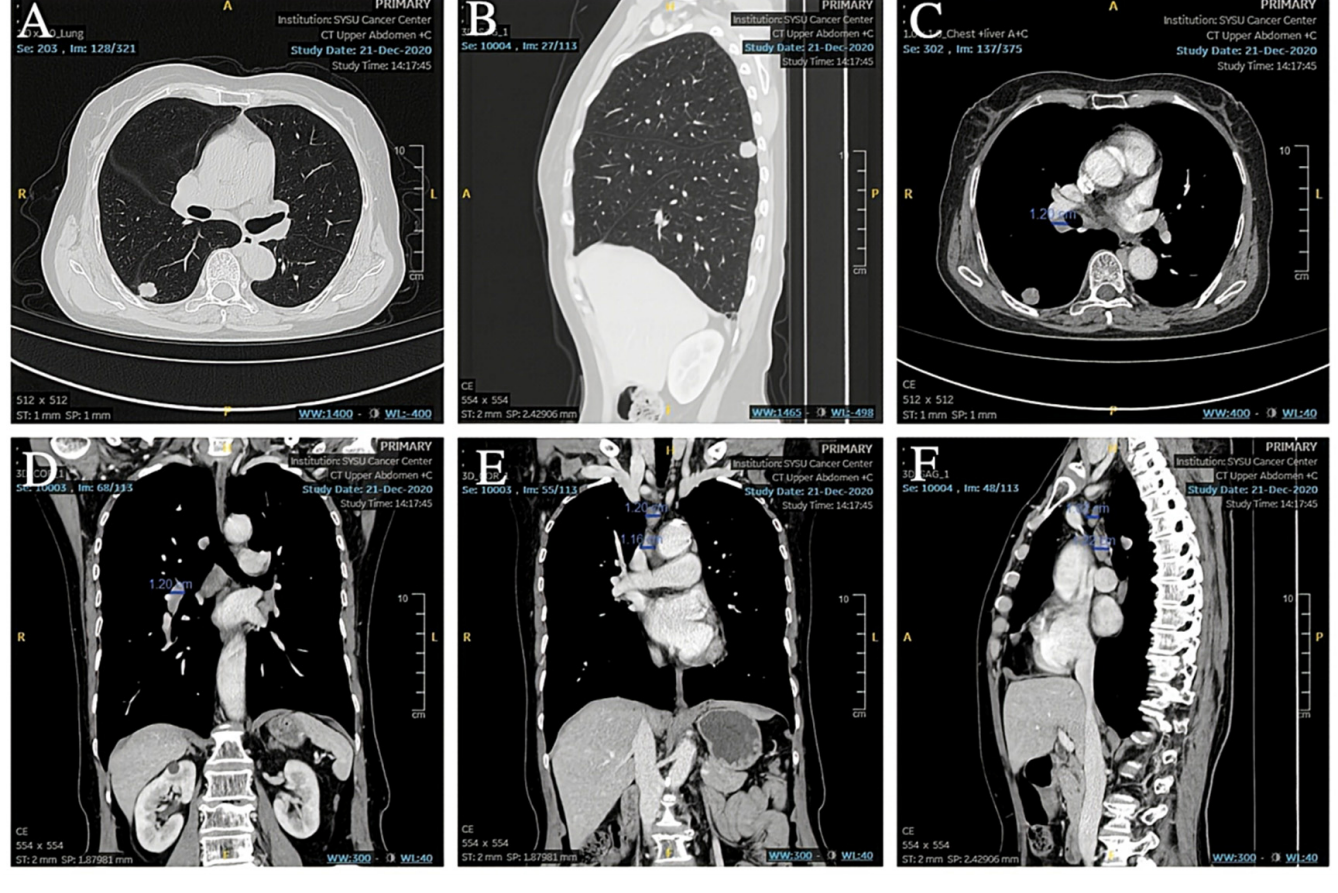
(**A**,**B**) Preoperative CT showed a space-occupying lesion in the lower lobe of the right lung. (**C**,**D**) Preoperative CT showed hilar lymph nodes with a short diameter less than 1 cm. (**E**,**F**) Preoperative CT showed other lymph nodes in the mediastinal region.

A 74-year-old female patient was referred to our hospital on 17 December 2020 due to an abnormality of the right lower lung detected on X-ray after a fall that injured her upper abdomen. She was never a smoker. Chest CT suggested that a nodule was seen in the dorsal segment of the lower lobe of the right lung, about 16 × 14 mm in size, with unclear borders, foliation, and short burrs at the margins, and significant enhancement was seen on enhancement scanning, which was adherent to the neighboring pleura (Figure 1A,B). No significantly enlarged lymph nodes in the hilar and mediastinal regions or larger lymph nodes with a short diameter of less than 1 cm (Figure 1C–F) were detected. No effusion was seen in the bilateral pleural cavity, and no thickening of the pleura was seen bilaterally. In addition, the cranial magnetic resonance and electron bronchoscopy did not reveal any obvious abnormality (PET-CT was not performed). On 23 December 2020, the patient underwent dorsal segmentectomy of the right lower lobe of the lung and mediastinal lymph node dissection. The histopathological report of the patient’s postoperative specimen showed heterogeneous cells arranged in nests, with little cytoplasm, high nuclear-to-pulp ratio, deep nuclear staining, and degeneration of individual nuclei accompanied by lamellar necrosis, which, combined with the immunohistochemical results, led to the diagnosis of a high-grade neuroendocrine carcinoma, consistent with small cell lung carcinoma. The five lymph nodes in the hilar and one in the mediastinal regions that were cleared intraoperatively showed no cancer metastasis, and the pathologic stage was pT2N0M0 IB.

**Figure 2:**
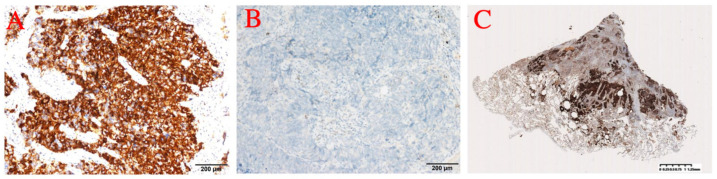
(**A**) ALK. (**B**) ALK-N. (**C**) ALK.

**Figure 3:**
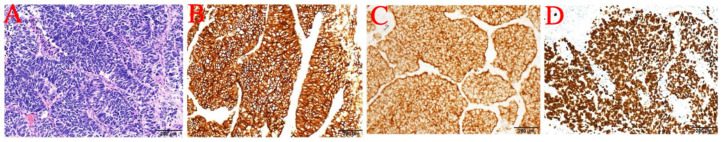
(**A**) 20× photo. (**B**) CD56. (**C**) Syn. (**D**) TTF-1.

Immunohistochemistry showed: ALK (D5F3) (+); ALK-N (−); CK (AE1/AE3) (+); CD56 (+); Syn (+); CgA (+); CK5/6 (−); CK7 (+); Napsin A (−); p40 (−); p63 (−); thyroid transcription factor-1 (TTF-1) (+); WT1 (−) (Figure 2 and Figure 3). The results of immunohistochemistry showed no non-small cell histologic incorporation.

**Figure 4:**
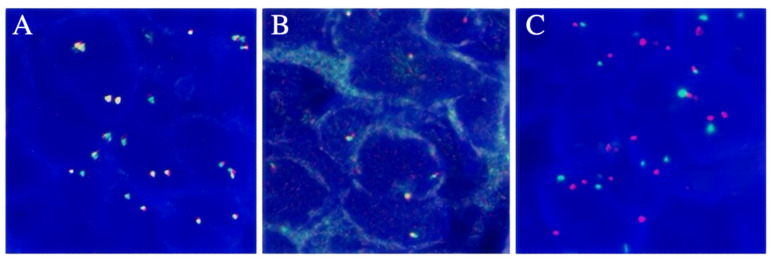
(**A**) The FISH result of ALK gene breakage test was negative. (**B**) The FISH result of ROS1 gene breakage test was negative. (**C**) The FISH result for C-MET gene amplification was negative.

**Figure 5:**
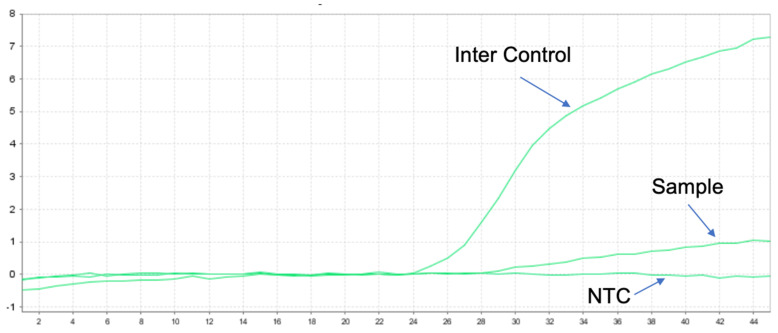
Positive results for v1–v5 of EML4-ALK by q-PCR for a total of nine known fusions.

**Figure 6:**
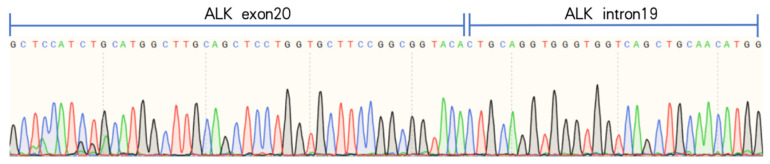
Designed EML4-ALK v5b specific primers, confirmed as EML4-ALK v5b isoforms by reverse transcription of RNA and Sanger sequencing, which showed the presence of v5b-specific ALK intron19 sequence.

We also performed genetic testing for the patient, and the report suggested epidermal growth factor receptor (EGFR) wild-type, negative ALK gene breakage test (Figure 4A), negative ROS1 gene (Figure 4B), and negative C-MET gene amplification (Figure 4C). NGS sequencing (Figure 4 and Figure 5): EML4-ALK gene fusion (E2; A20 V5b), mutation abundance was 2.41% (Figure 5 and Figure 6); TP53 (+) 53.81%; RB1 (+) 51.87%; TMB 11.2 muts/MB; PD-L1 TC >= 50%.

**Figure 7:**
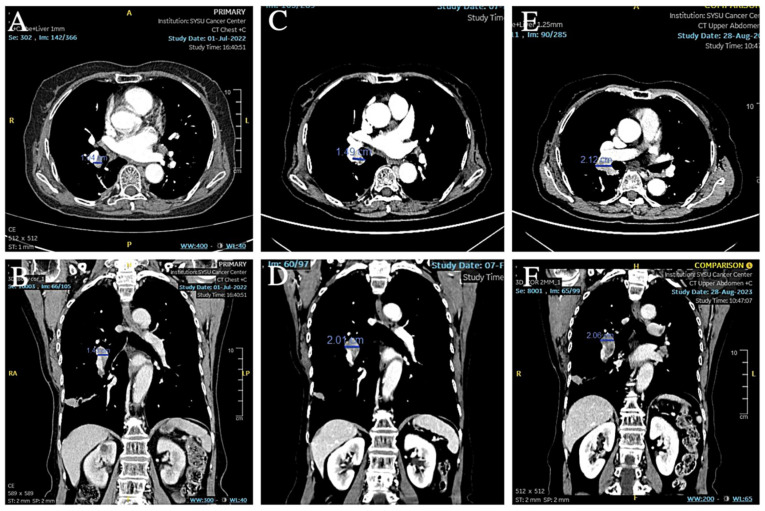
(**A**,**B**) CT showed that the short diameter of the right hilar lymph node had increased to 14 mm. (**C**–**F**) CT showed that the right hilar lymph node continued to increase in size and the short diameter increased to 21 mm.

**Figure 8:**
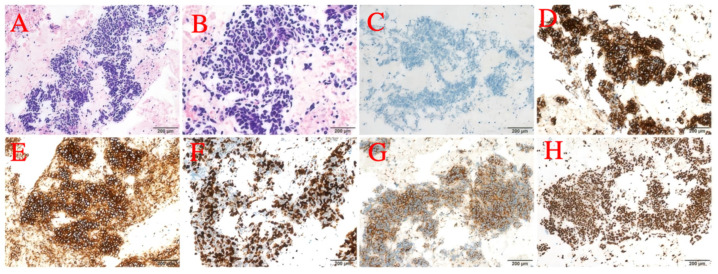
(**A**) 20× photo. (**B**) 40× photo. (**C**) ALK-N. (**D**) ALK. (**E**) CD56. (**F**) Ki-67. (**G**) Syn. (**H**) TTF-1.

From the end of the surgery to 2021-12-27 (12 months postoperatively), no abnormalities were found in the review imaging and no prophylactic treatment was performed. On 20 June 2022 (18 months postoperatively) when the CT was reviewed again, it was found that the right hilar lymph node was significantly enlarged compared to the previous one, with the short diameter increasing to about 14mm and with obvious enhancement, but still no corresponding treatment was performed (Figure 7A,B). During the period July 2022 to June 2023 (12 months), the patient’s CT results suggested that the lymph nodes were stable, and did not progress again until September 2023, and the patient refused treatment during this period. On 4 September 2023 (33 months after the operation), the CT examination was repeated and found that the multiple enlarged lymph nodes in both hilar regions had partially fused with each other, and the short diameter had increased to about 21 mm, with unclear boundaries and uneven enhancement, which was considered to have a high possibility of metastasis (Figure 7C–F). The patient was suspected of having recurrent metastases in the right hilar lymph nodes, so the patient received an ultrasonic bronchoscopic puncture biopsy (EBUS-TBNA), and the results of the biopsy showed that heterogeneous cells were seen in the right hilar lymph node fluid matrix and cell block sections, and the morphology was consistent with malignant tumor cells, which were suspected to be small cell carcinoma cells. Immunohistochemical results: ALK(D5F3) (+); ALK-N (−); CK(AE1/AE3) (+); TTF-1 (+); CD56 (+); Syn (+); CgA (+); and Ki-67 (about 80%+) (Figure 8). Immunohistochemistry results still showed no non-small cell histologic binding. Combined with the history and immunohistochemical results, this was consistent with small cell carcinoma relapse.

**Figure 9:**
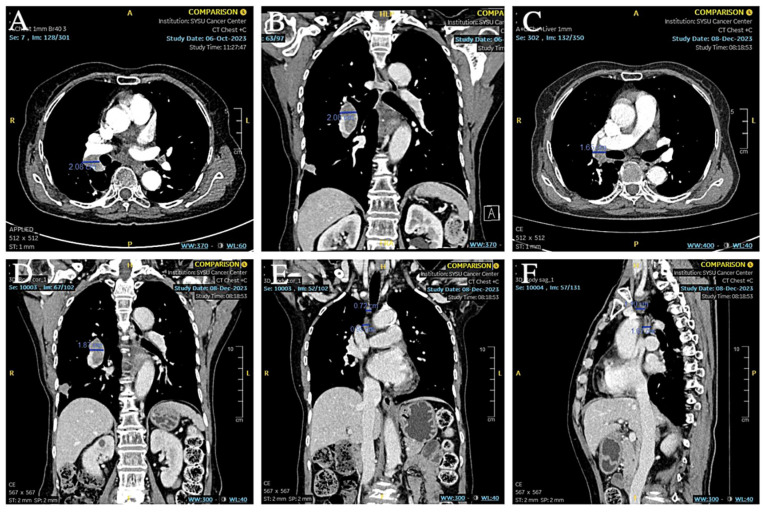
(**A**,**B**) CT showed that the hilar lymph nodes were smaller than before. (**C**–**F**) CT showed that the short diameter of hilar lymph nodes was reduced to about 19 mm, and other mediastinal lymph nodes were also reduced from the previous size.

Based on the patient’s medical history and the results of the puncture biopsy examination, we diagnosed this patient with hilar lymph node metastasis from SCLC combined with a rare EML4-ALK fusion gene mutation. Due to the patient’s refusal to undergo chemotherapy, ensartinib was chosen as the first-line agent for targeted therapy. The patient was given 225 mg of ensartinib (Ensacove) orally once a day for two weeks, and after two weeks of treatment, a repeat CT showed that multiple lymph nodes in the mediastinal region had partially shrunk compared to before. Since the patient developed symptoms of drug rash (CTCAE grade 4) within two weeks of the first dose, we adjusted the dose to 200 mg daily and continued treatment for two weeks. A repeat CT two weeks later (34 months postoperatively) suggested that multiple lymph nodes in the mediastinal region continued to shrink from the imaging two weeks earlier (Figure 9A,B). As this patient showed a trend toward improvement on imaging after a month-long course of ensartinib, we elected to continue treatment at ensartinib 200 mg daily for this patient. As of 8 December 2023 (36 months postoperative), the patient’s hilar lymph node had shrunk to a smaller short diameter of 19 mm (Figure 9C–F). The patient still received ensartinib and the cutoff time for documenting the condition in this case report was 31 January 2025 (49 months postoperative). The patient achieved a PFS of more than 12 months with ensartinib.

## 3. Discussion

Long-term survival for limited-stage SCLC has improved gradually over the past 30 years. However, the median survival time for limited-stage SCLC is only 15–20 months, and the 5-year survival rate is 15% or less, and most patients with SCLC recur and die of systemic metastases within 2 years [9]. This is a rare case of SCLC carrying a mutation in the EML4-ALK fusion gene. To our knowledge, this is the third case in China and the fifth case in the world of SCLC patients carrying ALK fusion mutations.

Among the five cases (Table 1), one case was detected in the component of SCLC combined with lung adenocarcinoma, and one case carrying PLEKHM2-ALK fusion mutation [10]. EML4-ALK fusion mutation in pure SCLC is extremely rare, with only three cases worldwide [10,11,12,13,14]. Previous SCLC cases carrying ALK fusion mutations are usually treated with chemotherapy as first-line treatment. In the case report by Li et al., adding an ALK inhibitor to chemotherapy resulted in an OS of more than 27 months, making this case the first reported case of a patient with SCLC with a fusion mutation in the ALK gene who had long-term benefit after treatment with an ALK inhibitor [10]. In another case report, the survival of patients with SCLC harboring an EML4-ALK fusion mutation was significantly improved after first-line treatment with crizotinib, demonstrating the potential of crizotinib for the treatment of SCLC accompanied by ALK fusion and further validating the potential of targeting the ALK gene mutation in such rare circumstances [13]. In fact, our case is the first of small cell lung cancer with confirmed EML4-ALK mutation after surgery.

**Table 1:** Summary of previously published case reports of patients with SCLC and ALK fusion [10,12,13,14].

Years	Articles Reported	Gene Mutation Types	Therapeutic Regimen	Therapeutic Effect Evaluation
2012	First Case of Combined Small-Cell Lung Cancer with Adenocarcinoma Harboring EML4-ALK Fusion and an Exon 19 EGFR Mutation in Each Histological Component.	EML4-ALK Fusion Mutation (SCLC combined with lung adenocarcinoma)	Lobectomy.	Loss to survival follow-up.
2013	An extremely rare case of small-cell lung cancer harboring variant 2 of the EML4-ALK fusion gene.	EML4-ALK Fusion Mutation	Platinum (carboplatin CBDCA/cisplatin CDDP) combined with etoposide (VP-16) chemotherapy.	Four months PFS and seven months OS.
2019	PLEKHM2-ALK: a novel fusion in small-cell lung cancer and durable response to ALK inhibitors.	PLEKHM2-ALK Fusion Mutation	Chemotherapy with carboplatin and etoposide; crizotinib added after identification of PLEKHM2-ALK rearrangement; crizotinib replaced with brigatinib and radiotherapy for brain metastasis.	More than 12 months PFS and 27 months OS.
2021	A Case of Small Cell Lung Carcinoma Harboring an EML4–ALK Fusion with Partial Response to Crizotinib.	EML4-ALK Fusion Mutation	Crizotinib.	PR.

According to previous reports, IHC was positive for ALK protein without EML4-ALK fusion mutation in SCLC patients [15], and previous reports also suggest that IHC should not be used as the only method to determine EML4-ALK fusion mutation, but it should be determined by a combination of multiple tests, and in addition, the genetic test can provide more personalized and targeted treatments. Actually, insufficient biopsy tissue in advanced-stage patients increases the difficulty of multiple tests. The patient in this case report had a post-operative clinically detected SCLC, which is usually characterized by the biobehavioral features of high aggressiveness, rapid progression, early metastasis, and easy recurrence [16]. For the patient reported in this case, sufficient tumor tissue was provided for multiple tests after surgery. After recurrence, the EML4-ALK mutation was confirmed again after re-biopsy using both IHC and NGS methods.

SCLC originates from neuroendocrine cells in the central airways, whereas LAdC originates from alveolar type II cells located on the alveolar surface [17]. Alveolar type II cells may be a common precursor of LAdC and SCLC [18,19,20,21]. Non-small cell lung cancer undergoes neuroendocrine transformation in the absence of TKI targets or other therapeutic approaches, as also demonstrated in previous studies [22]. The EGFR/TP53/RB1 triple mutation may increase the risk of SCLC transformation. However, SCLC transformation can also occur in ALK-positive patients [23]. In this case, the histologic component of non-small cells was not detected from the very first surgical specimen, and the patient could be discharged as a transformation of non-small cell lung cancer.

If SCLC is identified preoperatively according to previous guidelines, surgery will not be performed, but nowadays, for early stage SCLC (stage I-IIA), lobectomy should be preferred, with mediastinal lymph node dissection or systemic lymph node sampling if there is no lymph node involvement [7]. However, fewer than 5% of SCLC patients are true stage I-IIA patients, and most data on the role of surgery in SCLC comes from retrospective studies that have reported 5-year survival rates of 40% to 60% in stage I patients. Even for stage I SCLC, systemic chemotherapy is still recommended [24]. Based on data from previous SCLC cases harboring EML4-ALK gene fusion mutations, chemotherapy is necessary [25]. Nevertheless, the patient reported here refused to receive chemotherapy and received an ALK-TKI alone. By single-agent ALK-TKI ensartinib, the patient achieved a PFS of more than 10 months and an overall survival of more than 43 months, which is already far beyond the effectiveness of conventional treatment regimens. In addition, the patient only experienced the adverse effect of a drug-related rash. These results suggest that an ALK TKI alone may be a treatment option for advanced-stage SCLC patients with an EML4–ALK fusion mutation.

## 4. Conclusions

We report the fifth patient worldwide with SCLC harboring a fusion mutation in the EML4-ALK gene, who showed recurrent metastasis in the ipsilateral hilar lymph node at 18 months postoperatively and was diagnosed at 33 months postoperatively and was then started on treatment with the ALK-TKI ensartinib alone and achieved a PR efficacy with a PFS of more than 10 months and an OS of more than 43 months. An ALK TKI alone may be a treatment option for these patients. As there are so few cases of SCLC with ALK gene mutation, it is still not possible to summarize its characteristics and give a standard treatment plan as of now, and further studies are still needed. We continue to support the use of the ALK-TKI in this atypical case of SCLC and present our viewpoints on this case in the hope that it will provide a valuable reference for the treatment of such atypical cases in the future.

## Data Availability

Data are contained within the article.
